# Advances in clinical and translational research in endodontics: A comprehensive overview

**DOI:** 10.1590/0103-644020256723

**Published:** 2025-10-24

**Authors:** Renato Menezes Silva, Igor Bassi Ferreira Petean, Claudio Mendes Pannuti, Nathalia Vilela Souza, Yara Teresinha Corrêa Silva-Sousa, Giulio Gavini, Marco Antonio Hungaro Duarte, Carlos Estrela, Mike Reis Bueno, Jardel Francisco Mazzi-Chaves, Francisco Wanderley Garcia de Paula-Silva, Carla Renata Sipert, Fernanda Gonçalves Basso, Manoel Damião

**Affiliations:** 1 Department of Endodontics, University of Pittsburgh School of Dental Medicine, Pittsburgh, PennsylvaniaUSA.; 2 Department of Restorative Dentistry, School of Dentistry of Ribeirão Preto, University of São Paulo, Ribeirão Preto, SP, Brazil.; 3 Department of Stomatology, School of Dentistry, University of São Paulo, São Paulo, SP, Brazil.; 4 Faculty of Dentistry, University of Ribeirão Preto, São Paulo, Brazil.; 5 Department of Restorative Dentistry, School of Dentistry, University of São Paulo, São Paulo, SP, Brazil.; 6 Department of Operative Dentistry, Endodontics, and Dental Materials, Bauru School of Dentistry, University of São Paulo, Bauru, SP, Brazil.; 7 Department of Stomatologic Science, Federal University of Goiás, Goiânia, GO, Brazil.; 8 Department of Radiology, Faculty of Dentistry, São Leopoldo Mandic University, Campinas, SP, Brazil; 9 Department of Paediatric Dentistry, School of Dentistry of Ribeirão Preto, University of São Paulo, Ribeirão Preto, SP, Brazil.

**Keywords:** endodontics, translational research, clinical trials, diagnostic imaging, precision dentistry, molecular biology

## Abstract

Clinical and translational research play a decisive role in advancing evidence-based endodontics by bridging basic science with clinical applications. This narrative review provides a comprehensive overview of the current state and advances in clinical and translational research in Endodontics, addressing methodological foundations, diagnostic challenges, and emerging technologies. It highlights the importance of well-designed randomized controlled trials, robust outcome definitions, and the inclusion of patient-reported outcome measures. The integration of advanced imaging, particularly cone-beam computed tomography, has significantly improved diagnostic accuracy and treatment monitoring. Molecular biology techniques, including polymerase chain reaction and biomarker profiling, have expanded the understanding of endodontic microbiome, immune responses, and host factors related to treatment outcomes. Despite these advances, persistent limitations include diagnostic imprecision, lack of standardized criteria, and underutilization of biomarkers and omics data in clinical practice. The rise of precision dentistry, propelled by genomics, bioinformatics, and artificial intelligence, holds the potential to revolutionize endodontic care through personalized diagnostic and therapeutic strategies. Bridging existing gaps will require rigorous study designs, coordinated multicenter efforts, and the effective integration of molecular diagnostics, all of which are critical to advancing endodontic science and optimizing patient outcomes. Likewise, the full potential of translational science can be harnessed to reshape the future of endodontics.

## Resumo

A pesquisa clínica e translacional desempenha um papel decisivo no avanço da Endodontia baseada em evidências, unindo a ciência básica às aplicações clínicas. Esta revisão narrativa oferece uma visão geral abrangente e dos avanços na pesquisa clínica translacional em Endodontia, abordando fundamentos metodológicos, desafios diagnósticos e tecnologias emergentes. Destaca-se a importância de estudos clínicos randomizados bem controlados, definições de resultados robustos e a inclusão de medidas de resultados referidos pelos próprios pacientes. A integração de exames de imagem avançados, especialmente a tomografia computadorizada de feixe cônico, melhorou significativamente a precisão do diagnóstico e o monitoramento do tratamento. As técnicas de biologia molecular, incluindo a reação em cadeia da polimerase e investigação de biomarcadores, ampliaram a compreensão do microbioma endodôntico, das respostas imunológicas e dos fatores do hospedeiro relacionados aos resultados do tratamento. Apesar desses avanços, as limitações persistentes incluem a imprecisão do diagnóstico, a falta de critérios padronizados e a subutilização de biomarcadores e dados ômicos na prática clínica. A ascensão da Odontologia de Precisão, impulsionada pela Genômica, Bioinformática e Inteligência Artificial, tem o potencial de revolucionar o tratamento endodôntico por meio de estratégias diagnósticas e terapêuticas personalizadas. Para preencher as lacunas existentes, serão necessários projetos de estudo rigorosos, esforços multicêntricos coordenados e a integração eficaz de diagnósticos moleculares, todos essenciais para o avanço da ciência endodôntica e a otimização do tratamento endodôntico. Da mesma forma, todo o potencial da ciência translacional pode ser aproveitado para remodelar o futuro da Endodontia.

**Figure:**
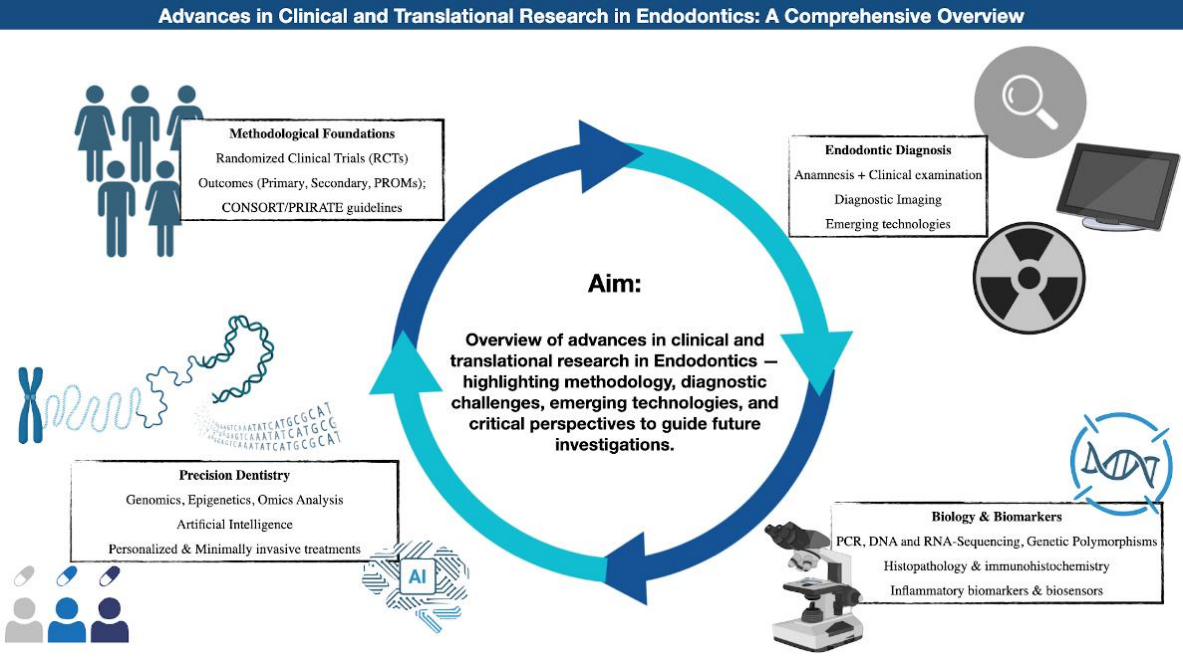

## Introduction

The goal of clinical and translational research is to build a base of scientific knowledge to address clinical needs. Translational science is considered the interaction of multiple disciplines promoting the integration of basic research, patient-oriented and population-based research, statistics, and informatics to improve population health ^1^ ^,^ ^2^. Translational research, also known as the "bench-to-bedside" enterprise, leads to the development of promising new treatments that can be used clinically or made marketable ^3^. Additionally, it aids in creating strategies to promote the adoption of best practices in clinical settings and informs policy development ^4^.

Translational research in dentistry plays a crucial role in enhancing patient outcomes and improving population health by linking basic science and pre-clinical research with clinical or community-based practice. For example, to support and validate the routine usage of a new dental drug, material, or technique, the translation process should involve several essential steps: fundamental science discovery, proposed human application, proven clinical testing and application, incorporation into clinical practice, and implementation at the population level ^5^.

However, scientific data on the topic reveal some obstacles in their development, which include variable and complex health conditions, diversity of practice settings, inefficient multidisciplinary collaboration, as well as avoidable research waste ^6^. Successful translation and implementation depend on standardizing the translation process and fostering collaboration among various stakeholders, including health policymakers, researchers, industry representatives, clinicians, and patients. Developing approval programs in dentistry that reasonably balance public protection and the speedy impact of emerging technology is another area that needs immediate attention. ^7^. Thus, considering that translating basic science to clinical research through well-planned translational research is key for meaningful advances in endodontics, basic researchers, clinician scientists, and clinicians should work as a team to achieve the set goals ^4^.

A robust translation process can lead to high-quality research and impact endodontics as a significant paradigm shift ^4^ ^,^ ^8^ ^,^ ^9^. Therefore, the purpose of this study is to provide a comprehensive overview of the current state and advances in clinical and translational research in endodontics, addressing methodological foundations, diagnostic challenges, and emerging technologies, in addition to performing a critical analysis of clinical and translational studies in Endodontic Research, aiming to support and guide future investigations in the area.

## Methodological Outline of Clinical Research

Randomized controlled trials (RCTs) represent the gold standard in evaluating the efficacy of interventions ^10^. In Endodontics, clinical research plays a crucial role in improving treatment outcomes, reducing patient discomfort, and enhancing overall care quality by investigating new medications, devices, and biomaterials. Like other medical fields, endodontic research requires rigorous methodology to ensure its findings are valid and applicable in practice ^11^. In this context, forming a research question and a corresponding hypothesis is the first step in the design of clinical research ^10^. The PICOS framework assists researchers in structuring their question by considering five key components: Population (P), Intervention (I), Comparison (C), Outcome (O), and Study design (S). This framework ensures the research question is specific, concise, and answerable, directing the entire protocol's development, from study design to sample size and statistical analysis.

Once the research question and hypothesis are set, investigators can draft the full study protocol. Strict adherence to the research protocol is important for maintaining the study's integrity and preventing bias, such as reporting bias and selective outcome reporting (SOR), which can impact the study's validity and reliability ^13^ ^,^ ^14^ ^).^ This adherence ensures that the study results are accurate and can be reliably applied in clinical practice.

One key element of the PICOS framework is the study design. In clinical trials, the most common designs include parallel, split-mouth, and crossover trials ^15^. Parallel trials are widely used but typically require larger sample sizes to achieve statistical power ^16^. In contrast, crossover trials involve each participant receiving multiple treatment sequences, thereby reducing the required sample size ^17^. This design is particularly suitable for evaluating interventions with temporary and reversible effects. However, in Endodontics, where procedures such as surgical and non-surgical treatments are definitive and irreversible, the use of crossover trials is generally inappropriate ^18^.

An alternative approach, specific to dental research, is the split-mouth design, which allows for intra-patient comparisons by applying different treatments to different areas within the same patient’s mouth ^18^. While advantageous in specific contexts, this design may be unsuitable for endodontic studies involving biologically active substances, such as intracanal medications, due to the risk of carry-over effects through systemic circulation or shared inflammatory pathways. In contrast, split-mouth designs may be appropriate for purely mechanical interventions, such as testing different instrumentation systems.

Ultimately, selecting the most appropriate study design in Endodontics depends on the specific research question and the biological or mechanical nature of the intervention being investigated.

Selecting appropriate participants is also critical. In endodontics, challenges arise with clustered data, such as participants with multiple endodontic teeth that need endodontic intervention. This scenario requires the use of advanced statistical approaches, such as multilevel or mixed-model analysis. This approach enables the clustering of teeth within individuals, thereby ensuring a more precise analysis of treatment effects ^19^.

Outcomes are variables used to address a research question and are categorized into primary and secondary ^20^. The primary outcome, being the most important variable of the study, typically dictates the calculation of sample size and the study's conclusion ^21^ ^,^ ^22^. In turn, secondary outcomes aid in interpreting the study's findings ^22^ ^,^ ^23^ and are often seen as exploratory, as the study's design does not primarily focus on them ^24^. Ideally, only one primary outcome should be chosen to reduce the risk of multiplicity ^25^. Multiplicity increases the chance of a Type I error, where treatment is mistakenly deemed superior due to a flawed null hypothesis rejection ^26^. To prevent this, the primary outcome must be pre-specified by defining its five levels: domain, specific measure, specific metric, aggregation method, and time-point ^27^. An example of a completely defined outcome would be: “change in the mean score of postoperative pain measured with the VAS scale 24 hours after baseline.” Equally important is the need to register the outcome before the study commences.

Preferably, primary outcomes should be clinically relevant, meaning that they represent a tangible effect for the patient ^20^. By definition, a clinically relevant outcome (CRO) “directly measures how a patient feels, functions, or survives” ^28^. These outcomes include objective or “hard” measures, such as mortality, and subjective measures, like quality of life ^20^. In Endodontics, tooth survival is a CRO, reflecting the primary goal of preserving natural teeth. The Periapical Index (PAI) ^29^ is another objective CRO. However, employing these clinically relevant outcomes can be challenging due to the extended time for their occurrence and the rarity of events. Overcoming these challenges requires larger sample sizes, multicenter studies, and more extended follow-up periods, which significantly increase research costs.

When CROs are not feasible, surrogate endpoints may be used as substitutes. Surrogate outcomes, as defined by Fleming in 2005 ^30^, do not directly measure clinical endpoints but rather serve as indicators of them. In medical research, blood pressure is a typical surrogate for cardiovascular events, predicting potential heart attacks or strokes. These surrogates provide quicker, more accessible measurements than actual clinical events. In endodontics, surrogate outcomes such as radiographic periapical healing and microorganism counts are used ^20^. However, they should only be used when CROs are unfeasible and must be validated to ensure they accurately reflect actual clinical benefits or risks.

Finally, Patient Report Outcome Measures (PROMs) are instruments, questionnaires, scales, or surveys designed to measure or capture outcomes reported by patients ^31^. PROMs provide valuable insights into patient morbidity and distress, serving as targets for therapeutic intervention and enhancing the quality of patient care through a comprehensive clinical decision-making approach (24). In the field of Endodontics, pain is a type of Patient-Reported Outcome (PRO) that can be measured, for instance, using a Visual Analog Scale (VAS). In this scale, patients are asked to rate pain on a scale ranging from 0 to 10 ^32^. Other examples of PROs include Oral Health-related Quality of Life (OHRQoL), measured using OHIP-14 ^33^, and patient satisfaction, assessed through a questionnaire ^34^. Including PROs in endodontic trials is important to develop a patient-centered, evidence-based approach in Dentistry, ultimately enhancing the value of treatment and research ^35^.

Upon completing a study and analyzing its data, authors have the responsibility to report their findings in a scientific journal accurately. Adhering to established guidelines for reporting clinical trials, such as the CONSORT (Consolidated Standards of Reporting Trials) guidelines, is of paramount importance to enhance the clarity, transparency, and reproducibility of research. The PRIRATE (Preferred Reporting Items for Randomized Trials in Endodontics) Guidelines ^36^, which are tailored to endodontic research, were created to improve the reporting of randomized clinical trials in Endodontics. Adhering to these guidelines ensures that research findings are communicated effectively to both practitioners and researchers, ultimately advancing the field of Endodontics.

### Importance of Structuring Diagnosis

The diagnostic process in Endodontics is a fundamental and challenging task, essential for establishing accurate therapeutic strategies. It is based on the identification of pathological conditions through a systematic analysis of clinical data obtained via anamnesis, physical examination, and complementary imaging techniques, including periapical radiographs and cone-beam computed tomography (CBCT) ^37^ ^,^ ^38^. The precision of this process directly affects treatment outcomes, requiring a structured, sequential, and highly strategic approach to mitigate errors arising from the inherent subjectivity of clinical information ^39^.

This process is grounded in three key pillars: semiogenesis, which addresses the origin and development of the patient’s signs and symptoms; semiotechnique, referring to the methodological approach employed in collecting clinical data; and propaedeutics, which encompasses the analysis, interpretation, and synthesis of information to formulate diagnostic hypotheses and comprehensive treatment plans ^40^.

Anamnesis represents the initial and indispensable step in the diagnostic workflow. Beyond its role in gathering pertinent clinical data, it establishes a therapeutic alliance between the clinician and patient. This process involves the systematic collection of personal identification, the chief complaint, the chronology of the current condition, relevant medical history, oral hygiene habits, and harmful behavioral patterns ^39^. In Endodontics, detailed pain characterization, including onset, duration, intensity, nature, and modulating factors, is critical ^41^. Furthermore, systemic diseases such as acute sinusitis may simulate endodontic symptoms. At the same time, conditions like metastatic carcinomas, particularly from the breast or lungs, may radiographically mimic periapical lesions, thereby underscoring the necessity of rigorous differential diagnosis ^42^ ^,^ ^43^.

The physical examination is structured into three sequential phases: general evaluation, extraoral examination, and intraoral examination ^40^. The general assessment involves observing the patient's somatic profile, posture, and behavioral patterns. Extraoral evaluation includes inspection and palpation of the head and neck regions, focusing on the skin, muscles, temporomandibular joint, and cervical structures to identify pathological signs such as edema, facial asymmetry, cutaneous fistulas, hematomas, or ecchymosis, often associated with pulp necrosis or maxillofacial trauma ^43^. The intraoral examination requires a systematic and comprehensive inspection of oral structures, including the labial and buccal mucosa, tongue, floor of the mouth, hard and soft palate, oropharynx, dentition, and periodontal tissues. The optimal execution of this phase mandates appropriate isolation, adequate lighting, and the use of retraction instruments to ensure precise observation of mucosal color, texture, and morphology ^40^.

Semiotechnical methods are integral to the clinical examination and comprise inspection, palpation, and percussion ^41^. Visual inspection facilitates the identification of morphological, colorimetric, and textural alterations in dental and soft tissues, often enhanced by optical aids such as magnification loupes, microscopes, and intraoral cameras ^44^. Palpation conducted digitally or with instruments assesses the consistency of soft tissue, the presence of swelling, fluctuation points, and dental mobility. Percussion evaluates the nociceptive response of dental structures to controlled mechanical stimuli, aiding in differentiating endodontic pathologies, typically associated with sensitivity to vertical percussion, from periodontal conditions or occlusal trauma, more frequently eliciting horizontal percussion sensitivity. Moreover, percussion may reveal ankylosis through the detection of characteristic hollow or metallic sounds ^41^.

Pulp sensibility testing constitutes a cornerstone of endodontic diagnosis. Thermal testing, particularly the application of cold stimuli, is the most widely utilized due to its simplicity, reliability, and clinical applicability ^45^. A positive response in vital pulps manifests as a brief, sharp pain, whereas necrotic pulps generally elicit no response. However, this method presents limitations in teeth with extensive restorations, elderly patients, or those exhibiting incomplete root formation ^39^. Although not routinely employed, the heat test can be a valuable adjunct in differential diagnosis, particularly in cases of pulpitis characterized by diffuse or radiating pain. In such scenarios, thermal stimulation may induce vasodilation, thereby amplifying nociceptive responses and facilitating the identification of inflamed pulpal tissue ^45^. This diagnostic approach is especially useful when responses to other tests are inconclusive, providing additional clinical insight into pulpal status. Electric pulp testing, historically prevalent, has seen a decline in contemporary use due to its relatively lower diagnostic precision ^45^. The cavity test, inherently invasive and irreversible, is reserved for cases where prior diagnostic modalities yield inconclusive results ^39^.

Emerging diagnostic technologies, notably pulse oximetry, offer non-invasive, objective assessment of pulp vitality based on hemoglobin oxygen saturation ^46^ ^,^ ^47^. Physiologically normal pulps exhibit oxygen saturation levels ranging from 70% to 94%, while inflamed or necrotic pulps present significantly reduced values ^47^. Despite its promising clinical applicability, demonstrated as effective even in traumatized teeth and those with periodontal involvement, the establishment of universally accepted reference values remains a subject of ongoing investigation within the literature ^47^ ^,^ ^48^.

Additionally, advanced optical technologies play a pivotal role in modern Endodontics. Magnification devices, such as Galileo and Kepler-type loupes, and operating microscopes, significantly enhance diagnostic accuracy by enabling precise visualization of fine structural details, including microcracks, fracture lines, and marginal restoration failures ^44^ ^,^ ^49^. The operating microscope, equipped with adjustable magnification, coaxial illumination, and ergonomic advantages, further facilitates the detection of subtle anatomical anomalies. Complementary diagnostic aids, including transillumination, bite tests, and the application of selective dyes, contribute to the identification of otherwise occult dental fractures and structural defects ^49^.

Therefore, endodontic diagnosis is an inherently multifactorial process that demands the integrated application of comprehensive anamnesis, meticulous physical examination, robust semiotechnical methodologies, and sophisticated technological adjuncts associated with diagnostic imaging. This rigorous and systematic approach is imperative to ensure diagnostic accuracy, clinical safety, and the achievement of predictable therapeutic outcomes ^37^ ^,^ ^50^.

### Importance of Diagnostic Imaging

The evolution of diagnostic imaging technologies has profoundly transformed endodontics, significantly enhancing diagnostic accuracy, clinical decision-making, and treatment outcomes. For many years, the primary supplementary resource to clinical examination was the analysis of the maxillofacial complex using conventional radiographic techniques, such as panoramic and periapical radiographs. However, achieving diagnostic accuracy has always been a challenge, as three-dimensional structures are assessed through two-dimensional images, often resulting in limited spatial information and difficulty in interpreting complex anatomical structures ^37^ ^,^ ^38^.

The introduction of cone-beam computed tomography (CBCT) has brought significant advancements to dentistry, particularly in endodontics ^37^ ^,^ ^38^ ^,^ ^51^ ^,^ ^52^. Unlike conventional radiography, CBCT offers high-resolution three-dimensional imaging, allowing accurate visualization of root canal systems, bone alterations, and periapical lesions. This technology provides detailed views in the axial, sagittal, and coronal planes, greatly enhancing the ability to detect periapical pathologies that may be obscured in 2D images.

A significant benefit of CBCT is its superior specificity and sensitivity for hard tissue assessment compared to 2D imaging ^38^ ^,^ ^51^. This enables comprehensive evaluation of complex root canal anatomies, root fractures, resorption defects, and periapical pathologies, such as apical periodontitis (AP), including in cases of lesions confined to cancellous bone that may not be visible on traditional radiographs ^38^ ^,^ ^52^.

The adoption of CBCT in endodontics has led to the development of advanced diagnostic indices. The Periapical Index (PAI), initially designed for 2D imaging, evolved into the CBCT Periapical Index (CBCTPAI), proposed by Estrela et al. (2008) ^38^, which incorporates volumetric analysis of lesions. CBCTPAI not only considers lesion diameter but also accounts for cortical bone expansion and destruction, offering a more robust framework for assessing AP ^52^ ^,^ ^53^. Additionally, a recent study demonstrated that combining CBCTPAI scoring with clinical evaluation can optimize decision-making in the management of persistent AP ^54^. For instance, cases classified with CBCTPAI scores of 4 and 5 in symptomatic patients were more frequently referred for endodontic surgery, underscoring the utility of volumetric CBCT assessments as an important diagnostic tool with clinical impact in treatment planning ^54^.

Further advancing this concept, the CBCT Periapical Volume Index (CBCTPAVI) was developed to provide precise volumetric measurements of lesions ^53^. CBCTPAVI allows clinicians to assess lesion volume and sphericity, critical factors in distinguishing between healing and persistent pathology ^52^ ^,^ ^53^. Moreover, evidence indicates that lesions with higher CBCTPAVI scores tend to exhibit reduced sphericity, reflecting a more irregular three-dimensional expansion pattern, which may complicate surgical access and influence healing dynamics ^55^. Evidence suggests that smaller lesions exhibit more predictable healing patterns, making volumetric CBCT assessment important for predicting treatment outcomes ^53^. Another study has shown that as the CBCTPAVI score increases, the sphericity of periapical lesions decreases, indicating that larger lesions tend to expand less uniformly and often elongate across one or more anatomic planes. The evaluation of these morphological characteristics by CBCT has implications for treatment planning, particularly in surgical endodontics, where the lesion’s shape may influence surgical access and lesion removal strategies ^55^.

Beyond diagnostic indices, CBCT technology has revolutionized case documentation and monitoring. Sequential CBCT imaging allows clinicians to track lesion progression or regression over time, providing valuable insights into treatment efficacy ^52^ ^,^ ^53^. This level of monitoring offers a distinct advantage over conventional radiography by enabling the detection of subtle anatomical changes not visible on 2D images. In this context, systematic reviews have shown that CBCT follow-up is more sensitive in detecting unhealed periapical lesions compared to conventional radiographs, particularly when assessing the quality and apical extension of root canal fillings ^56^.

A key technical advantage of CBCT is its ability to export images in the Digital Imaging and Communications in Medicine (DICOM) format, facilitating the integration and visualization of data from different systems using specialized software. These DICOM files can be converted to STL format for 3D printing, enabling the production of physical anatomical models useful in complex treatment planning ^38^ ^,^ ^52^. CBCT devices vary in sensor type, field of view (FOV), resolution, and software capabilities, allowing clinicians to select devices based on specific clinical requirements ^37^. This variability underscores the need for indication-oriented and patient-specific protocols to optimize diagnostic performance while adhering to the ALADAIP ("As Low As Diagnostically Acceptable, being Indication-oriented and Patient-specific") principle, improving the diagnostic accuracy and minimizing biological risks ^57^.

The growing demand for higher image quality and improved diagnostic tools has driven the development of advanced CBCT software solutions. For example, CDT Software (São José dos Campos, SP, Brazil) developed the e-Vol DX software, which standardizes image adjustments and enhances diagnostic accuracy across CT scans from various sources ^58^. This software supports high-resolution imaging with submillimeter voxel sizes, dynamic navigation through multiple planes, and adjustable parameters like slice thickness, contrast, and brightness. Additional studies have indicated that optimizing acquisition parameters such as field of view, voxel size, and milliamperage, together with appropriate artifact reduction tools, can further enhance the detection of fracture lines and complex anatomical features in endodontic scenarios ^59^ ^,^ ^60^.

Among the significant advancements within CBCT software is the implementation of artifact reduction algorithms, such as Blooming Artifact Reduction (BAR) ^58^ ^,^ ^61^. BAR significantly improves image quality by eliminating white contrast artifacts typically caused by metallic restorations or posts. A study demonstrated that the application of the BAR filter in e-Vol DX CBCT scans of teeth with metal posts showed no dimensional distortion when compared to direct micrometer measurements, underscoring its importance in ensuring diagnostic accuracy ^61^. However, it should be emphasized that CBCT imaging of endodontically treated teeth is prone to metallic artifacts caused by filling materials and intracanal medicaments, which can significantly impact diagnostic accuracy if not carefully managed ^62^ ^,^ ^63^ ^,^ ^64^. The successful application of BAR 0.5 in identifying separated instrument fragments in endodontically treated teeth was observed in a recent study ^65^. A color map algorithm, integrated into the post-processing software, was used to differentiate structures based on the molecular weight of the fragments, with red highlighting high-density areas. To ensure accuracy, image dynamic navigation across all planes was essential to mitigate beam hardening and radiation scattering. The color map facilitated the identification of separated instruments through dynamic, color-coded visualization. This approach proved more effective than periapical radiography, regardless of the obturation material, image view, or root canal analyzed ^65^.

Despite the considerable advantages of CBCT, proper education and training are essential for clinicians to correctly interpret scans and understand technical parameters such as voxel size, FOV, and software functionalities. Mastery of these elements ensures maximum diagnostic value while optimizing radiation exposure ^51^ ^,^ ^58^ ^,^ ^61^ ^,^ ^66^. Moreover, low-dose CBCT protocols have been shown to significantly reduce radiation exposure by up to 62% without compromising the accuracy of intraoperative detection of endodontic complications, highlighting their relevance in cases requiring multiple imaging acquisitions ^59^ ^;^ ^67^.

The emergence of CBCT as an essential diagnostic tool has not only improved clinical practice but also advanced protocols in clinical and translational research. Beyond its clinical utility, CBCT plays a pivotal role in bridging the gap between diagnostic imaging and translational research. Recent studies have demonstrated that its three-dimensional assessment of mineralized tissue alterations, such as pulp calcifications, apical periodontitis, lesion sphericity, besides complex root canal morphology, offers higher accuracy than conventional radiography, thereby facilitating patient stratification in clinical trials and influencing targeted therapeutic approaches in complex cases that demand high precision ^54^ ^,^ ^68^ ^,^ ^69^ ^,^ ^70^. This capability provides quantitative and qualitative data that enhance the design, execution, and interpretation of clinical trials, ultimately improving the external validity of translational investigations ^54^ ^,^ ^69^ ^,^ ^70^. Integrating such advanced imaging metrics into research protocols aligns diagnostic endpoints with biological and functional outcomes, strengthening the evidence base for minimally invasive strategies and biomaterial testing in endodontics ^68^ ^,^ ^70^.

In summary, CBCT has established itself as a cornerstone in modern endodontics ^71^. When appropriately indicated and optimized, CBCT decisively contributes to the accurate identification of periapical pathologies ^54^, root fractures ^60^, and complex root canal configurations, supporting evidence-based decision-making in persistent apical periodontitis ^54^. CBCT-derived indices like CBCTPAI and CBCTPAVI have become standard in longitudinal studies assessing periapical healing, lesion characterization, and treatment success ^38^ ^,^ ^52^ ^,^ ^53^.

Furthermore, the integration of artificial intelligence with CBCT is expected to improve diagnostic precision further, automate lesion detection, and standardize assessments in endodontic research ^58^ ^,^ ^61^ ^,^ ^66^. Its continuous development, coupled with advancements in dedicated software and AI-driven analysis, promises to elevate diagnostic confidence further and improve patient outcomes ^38^ ^,^ ^51^ ^,^ ^52^ ^,^ ^58^ ^,^ ^61^. Nevertheless, clinicians must remain aware of inherent limitations posed by beam hardening and metallic artifacts, which can compromise image interpretation, particularly in the presence of intracanal materials or high-density structures ^62^ ^,^ ^63^ ^,^ ^64^. In this context, the principles of ALADAIP should guide the selection of protocols, favoring low-dose and artifact-reduction strategies whenever feasible ^59^ ^,^ ^60^ ^,^ ^67^. In addition, the integration of magnetic resonance imaging has been proposed as a nonionizing alternative with comparable or even superior diagnostic performance for evaluating root canal anatomy, periapical lesions, and root fractures, particularly relevant for young patients or situations requiring repeated imaging ^72^. Therefore, the continued evolution of imaging technologies, optimization of acquisition parameters, and critical interpretation of data are essential to maximize diagnostic accuracy, in addition to enhancing the role of these tools in clinical and translational research, supporting the research protocols, and promoting advanced endodontic knowledge and practice while preserving patient safety.

### Collection of data and biological materials for clinical research in Endodontics

The collection of clinical data and biological materials is a fundamental step in the design and execution of endodontic research. Ensuring accuracy, reproducibility, and ethical management in these processes is essential for generating high-quality evidence, advancing scientific knowledge, and ultimately enhancing patient care.

In clinical research, the data collection process typically begins with the acquisition of comprehensive demographic and medical information, including patient age, sex, systemic health conditions, medication use, and dental history ^37^. A detailed record of the patient’s chief complaint, pain characteristics, clinical examination findings, and radiographic assessments is essential for ensuring a robust dataset that reflects the complexity of endodontic diagnosis and treatment ^38^ ^,^ ^51^.

In addition to clinical and imaging data, the collection of biological materials is increasingly incorporated into endodontic research to investigate microbial, cellular, molecular, and histopathological aspects of endodontic infections and healing processes. Standard biological samples include dentin shavings, root canal exudates, dental pulp, necrotic pulp tissue remnants, periapical granulomas, cystic tissues, and other root canal content, including microorganisms. Furthermore, biological materials are utilized for immunohistochemical analyses and biomarker detection, using, for example, Western blotting or ELISA (Enzyme-Linked Immunosorbent Assay), providing insights into the host immune response, inflammation, and tissue repair processes in periapical diseases. The identification of cytokines, growth factors, and other molecular mediators within periapical tissues contributes to the understanding of enteropathogenic events and the development of regenerative strategies and novel therapeutic approaches ^73^.

All clinical research involving human participants, including studies in Endodontics, must rigorously follow internationally recognized ethical standards, primarily the Declaration of Helsinki ^74^. This declaration emphasizes the protection of human dignity and autonomy, prioritizing participants' rights over the interests of science or society.

The collection of biological materials necessitates the acquisition of explicit informed consent, obtained through a transparent and comprehensive communication process. Participants must be thoroughly informed regarding the study’s objectives, the specific nature of the biological materials being collected, the procedures for processing and storage, and the potential for future use in additional research endeavors ^75^ ^,^ ^76^. Furthermore, any foreseeable risks, discomforts, or potential direct benefits associated with participation must be clearly disclosed ^77^.

Ethical obligations extend well beyond the procurement of consent, encompassing stringent measures to safeguard privacy and the confidentiality of personal data, given the inherently sensitive nature of biological materials. Robust mechanisms, such as anonymization or secure coding of data, are essential to protect participant identity. Additionally, participants retain the unequivocal right to withdraw consent at any stage without any repercussions to their ongoing clinical care ^78^ ^,^ ^79^.

All research protocols involving biological materials must be rigorously reviewed and approved by an Institutional Review Board or Research Ethics Committee. This process ensures strict adherence to established ethical guidelines, the maintenance of an acceptable risk-benefit ratio, and the mitigation of potential harm ^74^ ^,^ ^77^. Special ethical considerations are mandatory when involving vulnerable populations, including minors and individuals with cognitive impairments, necessitating the acquisition of legal guardian consent in conjunction with participant assent whenever feasible ^80^.

The governance of biological materials, whether for genetic research, biobanking, or long-term storage, demands unequivocal transparency regarding storage duration, conditions of future use, policies for data sharing, and clear directives concerning the disposal of samples upon study completion or at the participant’s request ^81^ ^,^ ^82^. Failure to comply with these ethical imperatives compromises research integrity, invites legal repercussions, and erodes public trust. Conversely, rigorous compliance fosters transparency, fortifies participant autonomy, and sustains the scientific and ethical integrity of endodontic research ^74^ ^,^ ^77^.

Another important point during the collection of biological materials is that standardization in sample collection, storage, and processing is essential to maintain the integrity of the samples. Samples are typically stored in sterile containers under controlled temperatures, often at −80°C for long-term preservation, to prevent degradation of nucleic acids, proteins, and other biomolecules ^73^ ^,^ ^75^.

Thus, careful collection, storage, and management of clinical data and biological specimens, providing the formation of a biobank, is pivotal for contemporary endodontic research. This integrative approach not only enhances the understanding of endodontic pathology and treatment outcomes but also paves the way for translational research focused on precision dentistry.

### Molecular Biology Tests

Molecular biology is the science field that investigates the molecular foundations of biological events. Gene expression and regulation, especially DNA replication, transcription, and protein synthesis, are included in this branch of Biology ^83^. Developed by Kary Mullis in 1983, the polymerase chain reaction drastically impacted biological research, thus earning Mullis the Nobel Prize in Chemistry in 1993. This laboratory technique provides the exponential replication of DNA, allowing robust and sensitive analyses of the genetic material of a plethora of distinct sample sources. This method is currently considered one of the most relevant advances in molecular technology ^84^.

Studies using molecular biology approaches have been published in the field of endodontics since the 1990s. The first study aimed to investigate inflammatory genes in chronically inflamed tissues, including apical periodontitis, using mRNA analysis ^85^ ^,^ ^86^. Therefore, due to the sensitivity and specificity of this methodological tool, it was employed for studies regarding oral microorganisms, especially those colonizing root canals in teeth with pulpitis and pulp necrosis ^87^ ^,^ ^88^. Since then, numerous studies have been conducted to elucidate the role of distinct bacteria in apical periodontitis ^84^, particularly focusing on the detection of uncultivable microorganisms.

Based on the fast knowledge enrichment in endodontic microbiology achieved by molecular methodology in clinical investigations, this research tool was found helpful in studies aiming to analyze bacterial reduction along the root canal treatment procedures ^89^. Many studies further investigated specific issues in endodontic treatment by using molecular biology approaches ^90^ ^,^ ^91^ ^,^ ^92^ ^,^ ^93^ ^,^ ^94^ ^,^ ^95^. Besides PCR, other molecular methodologies have been proven as effective as it is in clinical microbiology studies, such as checkerboard DNA-DNA hybridization, microarray ^92^, and next-generation sequencing ^91^, among others.

Recently, molecular studies have focused on genetic factors that may be associated with failed endodontic treatments or resistance to the development of pulp and periapical inflammation. Research on gene polymorphisms may enhance our understanding of the mechanisms underlying the onset and persistence of apical periodontitis ^96^ ^,^ ^97^ ^,^ ^98^ ^,^ ^99^ ^,^ ^100^ ^,^ ^101^. Taken together, we should recognize that molecular tests are a useful methodological tool for the translation of experimental in vitro and in vivo research findings to clinical studies, especially for understanding disease mechanisms, which in turn contributes to endodontic treatment improvement ^84^ ^,^ ^94^ ^,^ ^102^ ^,^ ^103^.

### Histopathological Investigations

Besides the advances of molecular biology in demonstrating the pathways and molecules involved in the etiopathogenesis of endodontic lesions, the histopathological analysis remains one of the most reliable and demonstrative methods to determine the final diagnosis of periapical lesions. It may also be a valuable tool for the demonstration of diagnostic and therapeutic markers ^104^. To date, 75% of studies evaluating periapical lesions have used conventional histological analysis to demonstrate results, while 66% of these studies have also used immunohistochemistry techniques to achieve this purpose ^105^.

These methods include conventional processing and staining using hematoxylin and eosin, as well as Masson's Trichrome, which may provide relevant data regarding lesion organization (presence or absence of a cystic epithelium), presence, density, and type of inflammatory population, as well as the organization of extracellular matrix, such as collagen fibrils ^106^. Moreover, other methods using histological specimens include the identification of protein epitopes (antigens) using specific antibodies for fluorescence microscopy or immunohistochemistry ^104^.

It is important to remember that the correct collection, storage, preparation, and processing of samples for histological analysis are crucial for the obtention of optimized and standardized results. Regarding this concern, several points warrant consideration. Firstly, the fixation solution must be selected based on further analysis. Formaldehyde 4% (also known as formalin 10%) is the primary choice for this process, having presented satisfactory results. However, determining some low-expressed proteins may require weaker chemical agents, such as paraformaldehyde. Time of fixation is also an important factor: researchers have demonstrated that samples should be immersed in fixation solution (20x w/v) immediately after acquisition, as significant time-dependent protein degradation and loss of epitopes were detected after 2 hours, with complete epitope loss when samples were fixed after 6 hours ^107^.

Another method’s jeopardy is the period of fixation. According to previous investigations, the standard time-point for fixation is 24 hours, and the maintenance of samples in fixation solution for more than 4 hours may also result in loss of samples' adherence to histological slides, as well as loss of protein epitopes. Therefore, the proper protocol must also consider the sample's dimensions ^107^.

Collection and preparation may also influence its fixation and staining. First, the obtention of a representative volume of tissue is mandatory for a reliable analysis. In addition, for periapical lesions, the careful handling of the lesion and storage is critical to determine, for example, whether it may be classified as a periapical cyst or granuloma. On the other hand, studies regarding the evaluation of the dentin-pulp complex, such as materials testing, may be handled differently. Studies demonstrate that separating the crown and root parts before fixation may inhibit tissue shrinkage and poor fixation ^104^.

Histopathological analysis enables the evaluation of materials and protocols not only in pre-clinical studies but also for clinical trials. It may provide complete information regarding the effects of these strategies on the dentin-pulp complex, periodontal, and apical tissues. Therefore, the use of histological analysis remains one of the most reliable and feasible investigation methods for diagnostic and therapeutic studies in endodontics, as its results demonstrate the effects of different protocols on complex and organized tissues and may provide more translational data for the establishment of new materials and techniques.

### Biomarkers involved in Endodontic pathologies

Currently, the diagnosis of pulp inflammation is based on patient history, clinical examination, and radiographic assessment, methods that have remained essentially unchanged for over a century. However, the reliability of these clinical tests accurately reflecting the pulp's histopathological condition remains a subject of ongoing debate. A literature review summarized the available evidence regarding the diagnostic accuracy of clinical signs, symptoms, and current tests used to determine pulp status ^108^. The authors concluded that the overall evidence is insufficient to support the accuracy of these tests, even when used in combination. Consequently, current diagnostic procedures do not reliably identify the inflammatory status of the pulp, which may significantly compromise clinical decision-making processes. This is particularly relevant in choosing between vital pulp therapies and root canal treatment, both of which critically depend on accurate pulp diagnostics.

According to the National Library of Medicine, the Medical Subject Headings (MeSH) definition of a biological marker is a measurable and quantifiable biological parameter that serves as an indicator for health and physiological assessments ^109^. Molecules expressed within the tissue inflammation cascade can serve as diagnostic biomarkers of inflammatory processes. Some studies suggest that the dental pulp should not be considered an isolated entity encapsulated within a rigid environment but rather a reactive tissue capable of releasing its biological products into the external environment ^110^ ^,^ ^111^ ^,^ ^112^. Indeed, research has demonstrated that pulpal events can be reflected by measurable levels of protein markers correlating with pulp symptoms in various fluids, including pulpal blood, dentinal fluid, periapical fluid, gingival crevicular fluid, and even saliva, enabling non-invasive assessment ^110^ ^,11,^ ^112^.

In periodontology, biomarkers in oral fluids such as saliva or gingival crevicular fluid are routinely employed to detect the presence and progression of periodontitis. For example, matrix metalloproteinases (MMPs), particularly MMP-8 and MMP-9, have been identified as key biomarkers associated with soft tissue degradation in periodontal pockets ^113^. Notably, periodontal and pulpal inflammation share several pathophysiological characteristics; both initially involve soft tissue inflammation driven by microbial infection and later progress to bone resorption. Therefore, it is plausible that both pathologies express common biomarkers. In this context, MMPs have been proposed as potential biomarkers for both pulpal and periodontal diseases. However, the application of molecular diagnostics in pulpal disease has not yet been translated into routine clinical decision-making.

Clinical studies investigating the presence of potential biomarkers associated with pulpal inflammation are still emerging ^110^ ^,^ ^111^ ^,^ ^112^. The clinical significance of identifying such biomarkers for pulp inflammation justifies not only further primary research but also comprehensive critical or systematic reviews of the published evidence. Systematic reviews of well-designed randomized controlled trials are regarded as the gold standard for clinical evidence ^114^, as the development of clinical guidelines based on systematic reviews contributes to improving the quality of dental and medical care for the general population. These guidelines provide evidence-based recommendations to assist clinicians and patients in making informed healthcare decisions for specific conditions ^115^.

A recent systematic review critically evaluated the available data regarding biomarkers identified in pulp tissues classified as either healthy or inflamed ^110^ ^,^ ^111^ ^,^ ^112^. The findings demonstrated the involvement of various Toll-like receptor (TLR)-induced chemotactic molecules, named chemokines, including IL-8, CXCL-10, RANTES, and eotaxins, among others. As previously reported, under normal conditions, only a minimal presence of immune cells is observed in the dental pulp ^116^. Upon infection, immune cells are recruited, and soluble bacterial products, along with arachidonic acid pathway metabolites and chemokines, act as chemoattractants for leukocytes ^117^. The exponential increase in infiltrating leukocytes is accompanied by elevated lysosomal enzyme activity, which contributes to tissue degradation. Proteases such as elastase and MMPs degrade elastin and proteoglycans, leading to irreversible pulp tissue damage. Additionally, the expression of inflammatory mediators such as prostaglandin E2 (PGE2) and bradykinin promotes vasodilation and increased microvascular permeability by binding to their respective receptors, thereby inducing cytoskeletal rearrangements ^110^ ^,^ ^111^ ^,^ ^112^ ^,^ ^118^.

Concurrently with the destructive effects of leukocyte infiltration, these immune cells also possess reparative capabilities. This is mediated through the release of factors such as vascular endothelial growth factor (VEGF), transforming growth factor-beta (TGF-β), and granulocyte-macrophage colony-stimulating factor (GM-CSF), which contribute to extracellular matrix remodeling, endothelial cell proliferation and migration, and the formation of differentiated capillaries ^119^. Furthermore, the increased expression of beta-defensins in inflamed pulp plays an essential role in innate host defense against bacterial invasion, enhances adaptive immune responses, and exhibits chemotactic activity. Additionally, anti-inflammatory mediators, such as tissue inhibitors of metalloproteinases (TIMPs), are also upregulated during pulpal inflammation ^120^. Therefore, the balance or imbalance between tissue-destructive molecules like proteases and tissue-inductive molecules such as VEGF may serve as a diagnostic or prognostic tool for endodontic interventions ^110^.

Despite this progress, a significant challenge remains: the development of methods to enable reliable, chairside quantification of these biomarkers in clinical settings. Biosensors have been described as devices designed for this purpose ^121^. Ideally, point-of-care diagnostic tests for biomarkers in endodontics should be simple, cost-effective, and rapid. For instance, lateral flow membrane-based immunoassays, similar to those developed for periodontology ^122^, could be adapted to detect individual biomarkers with predictive value for vital pulp treatment.

For more complex diagnostic needs, advanced technologies such as Lab-on-a-Chip and Lab-on-a-Disk utilize microfluidics and electronic engineering to analyze metabolites and other molecules from small biological samples ^123^ ^,^ ^124^. These technologies can replace centralized laboratory diagnostics, providing real-time clinical data that are microbiological, immunological, and metabolic in nature. Biosensors can detect nucleic acids, microbial components, glucose, oxygen, carbon dioxide, pH, and more with high sensitivity.

Consequently, the search for a single "gold standard biomarker" may be overly simplistic, particularly in the context of complex disease states. Instead, the exploration of comprehensive biosignatures represents the future of endodontic diagnostics. Ultimately, this approach may lead to the development of simplified yet highly accurate diagnostic assays that offer significant benefits for both clinicians and patients ^112^.

### Future perspectives: Precision Dentistry

Individual biological and genetic factors may account for one-third of all determinants of health ^125^. The most well-studied impact of precision medicine on health care today is genotype-guided treatment ^126^. A good example is the genomic profiling of tumors that results in targeted therapy plans for patients with breast or lung cancer ^127^.

Lifestyle factors such as poor dental hygiene are known to increase the risk for caries, periodontal disease, pulpitis, and apical periodontitis, but genes are also thought to play a role. Oral health and genetic data collected from over 500,000 participants identified 47 areas of the genome linked to dental caries ^128^. Individualized or personalized dentistry is an innovative and emerging approach for disease treatment and prevention that considers individual genetic variability, environment, and lifestyle for each person. The field has evolved to recognize how the intersection of multi-omics data combined with medical history, social/behavioral determinants, and environmental knowledge precisely characterizes health and disease states, resulting in possible therapeutic options for affected individuals ^126^.

The diagnosis and treatment of oral diseases are also becoming more accurate and specialized. Researchers are now identifying the molecular fingerprints of various oral diseases. Precision dentistry enables doctors to predict more accurately which treatment and prevention strategies for a particular disease will be effective in specific groups of people. It contrasts with traditional medicine or dentistry, where one-size-fits-all approaches are used, in which disease treatment and prevention strategies are developed for the average person, with less consideration for individual differences ^126^ ^,^ ^129^ ^,^ ^130^.

DNA, RNA, and proteins are responsible for orchestrating all the cellular functions in our body. Wound healing and tissue regeneration are complex processes that involve cellular communication and activation of signaling molecules, extracellular matrix components, remodeling enzymes, cellular adhesion molecules, growth factors, cytokines, and chemokines ^131^. In recent years, the knowledge generated by decoding the human genome has allowed groundbreaking genetic research to better understand genomic architecture and heritability in healthy and disease states ^132^.

It has become clear that individual variability may play a role in the response to different dental treatments ^133^. Host factors such as genetic polymorphisms may affect host physiology. Dental caries, periodontal disease, pulpitis, and apical periodontitis (AP) are collectively derived from dynamic interactions between host and microbial factors, where a complex inflammatory immune response mediates tissue destruction ^131^. However, the uncertainty of a lesion’s progressive or stable phenotype complicates understanding of the cellular and molecular mechanisms triggering lesion activity ^131^. The identification of key molecules for apical periodontitis has the potential to improve currently available therapies ^131^ ^,^ ^133^. Recently, a study identified novel genes and loci contributing to disease development and progression, as well as specific contributions to AP risk in men and women. The study also demonstrated that some systemic conditions are significantly associated with AP risk, providing strong evidence for host-mediated effects on AP susceptibility ^134^.

Recent advances have also highlighted the importance of epigenetic mechanisms, such as DNA methylation and microRNAs, in regulating gene expression in oral diseases. These epigenetic modifications can influence inflammatory responses and bone remodeling without altering the underlying DNA sequence, thereby contributing to disease susceptibility and progression ^135^ ^,^ ^136^ ^,^ ^137^.

Given the advances in research, dentists need to consider the potential for individualized dentistry, customized for each patient, where biological information acquired through genetic testing or biomarker profiling may directly influence diagnosis, treatment, and, ultimately, pulpal and periapical disease prevention. The discovery of novel target genes and pathways involved in pulpal and periapical disease and host response may point to promising new options for endodontic diagnosis. At the same time, future research on biologic agents as immune modulators may lead to improved, precision-based therapies. However, identifying independent phenotype-associated genes with high reliability remains challenging, especially for complex diseases and conditions ^134^.

Identifying shared genetic determinants between oral health and general health would be a great accomplishment. It may reduce oral care expenditures and, most importantly, improve people's lives ^125^.

### Critical Analysis of Clinical Studies in Endodontic Research

Clinical research in endodontics has experienced significant growth over the past decades, progressively shifting from anecdotal case reports to more structured, evidence-based methodologies. Despite this advancement, a critical examination reveals persistent methodological limitations that continue to affect the reliability, validity, and applicability of findings within the field. A considerable proportion of endodontic clinical studies still suffer from design weaknesses, including small sample sizes, inadequate randomization, lack of blinding, and limited follow-up periods ^114^ ^,^ ^115^. These issues heighten the risk of bias, particularly selection, performance, and detection bias, thereby compromising the internal validity of many studies. Even among randomized controlled trials, widely regarded as the gold standard in clinical research, such methodological flaws remain common and concerning ^22^.

A notable concern in endodontic clinical research is the lack of standardization in diagnostic criteria, especially regarding pulpal and periapical status. Conventional diagnostic tools, such as pulp sensibility tests based on thermal or electrical stimuli, are well-documented to have poor correlation with the histopathological condition of the pulp ^39^. This diagnostic uncertainty introduces potential misclassification of cases, particularly in studies evaluating the outcomes of vital pulp therapy, regenerative procedures, or root canal treatments. The absence of objective, biologically driven diagnostic tools continue to undermine the robustness of research outcomes in this domain ^112^.

An additional challenge is the heterogeneity in outcome definitions and assessment parameters across studies. While some investigations focus on radiographic healing as the primary outcome, others assess tooth survival, absence of clinical symptoms, or even subjective parameters like patient discomfort ^138^. This lack of consistency hinders the comparability of results between studies. It limits the capacity to perform meaningful meta-analyses, ultimately impeding the development of strong, generalizable evidence for clinical guidelines. Furthermore, the underrepresentation of patient-centered outcomes, such as quality of life, treatment satisfaction, and long-term functionality, restricts the real-world applicability of current research findings. Historically, endodontic research has prioritized biological and radiographic success, often neglecting how these outcomes translate into patient-perceived benefits ^115^.

Despite advancements in molecular diagnostics, bioinformatics, and imaging technologies, the integration of biomarkers and point-of-care diagnostic tools into clinical endodontic research remains scarce. Incorporating biomarkers, such as inflammatory mediators detectable in pulp tissue, periapical exudate, crevicular fluid, or saliva, has the potential to enhance diagnostic precision and prognostic accuracy ^112^ greatly. However, few clinical trials to date have successfully integrated these technologies into their methodologies, representing a critically missed opportunity for innovation in the field.

Ethical considerations and reporting transparency are additional concerns. Many clinical studies in endodontics inadequately document ethical approvals, participant consent processes, and comprehensive risk-benefit analyses ^26^. Moreover, adherence to standardized reporting frameworks such as CONSORT remains inconsistent, further limiting the ability of researchers and clinicians to appraise and replicate findings critically. The absence of rigorous reporting undermines transparency, compromises research reproducibility, and reduces the overall quality of published evidence.

These methodological and ethical limitations further hamper the translation of research findings into clinical practice. Although some studies have contributed to the development of evidence-based guidelines, particularly in areas like nonsurgical root canal treatment ^135^, significant gaps persist between research and clinical reality. Critical fields such as regenerative endodontics, biomaterials testing, and minimally invasive endodontic approaches remain dominated by preclinical studies, with limited high-level clinical evidence to support widespread clinical adoption.

In conclusion, while endodontic clinical research has made considerable progress, it remains constrained by persistent challenges related to study design, diagnostic inconsistency, heterogeneity of outcomes, lack of patient-centered measures, and insufficient use of emerging technologies. Addressing these limitations requires a paradigm shift toward multicenter, well-powered randomized controlled trials with standardized diagnostic protocols, consistent outcome measures, and greater incorporation of molecular diagnostics and biosensors. Improving ethical rigor and adopting comprehensive reporting standards are equally essential to enhance the scientific credibility, clinical relevance, and translational impact of future research in Endodontics.

## Conclusions

Recent advances in clinical and translational research in Endodontics have significantly enhanced diagnostic precision, treatment planning, and therapeutic outcomes by integrating robust methodological frameworks, advanced imaging modalities, molecular biology techniques, biomarker profiling, and principles of precision dentistry. Well-designed randomized controlled trials, standardized diagnostic criteria, and the inclusion of patient-reported outcomes are essential to strengthen the scientific evidence base and improve patient-centered care. Innovations such as CBCT and their derived indices, molecular diagnostics, and omics-driven approaches have expanded the understanding of disease mechanisms and host response for different therapies, paving the way for personalized treatment strategies. However, persistent challenges, including diagnostic heterogeneity, underutilization of biomarkers, and limited multicenter collaboration, still hinder the complete translation of scientific discoveries into everyday clinical practice. Bridging these gaps will require coordinated efforts among researchers, clinicians, industry, and policymakers, supported by ethical rigor and transparent reporting. By harnessing the full potential of translational science, Endodontics can progress toward a future defined by precision, minimally invasive interventions, and improved long-term outcomes for patients.
