# Errata

**DOI:** 10.1590/0103-644020256723er

**Published:** 2025-12-01

For the article published as:

Silva RM, Petean gor BF, Pannuti CM, Souza NV, Silva-Sousa YTC, Gavini G, et al.. Advances in clinical and translational research in endodontics: A comprehensive overview. Braz Dent J [Internet]. 2025;36:e23-6723. Available from: https://doi.org/10.1590/0103-644020256723

Is changed and completed the authors' names

Where is read:

Mike Bueno

Carla Sipert

Fernanda Basso

Must be read:

Mike Reis Bueno

Carla Renata Sipert

Fernanda Gonçalves Basso

For the article on internet site

Where is read:

gor Bassi Ferreira Petean

Must be read:

Igor Bassi Ferreira Petean

